# An investigation into internal exposure management needs for nuclear medicine practitioners and temporary visitors through I-131 internal dose assessment: Focusing on large hospitals in South Korea

**DOI:** 10.1371/journal.pone.0209244

**Published:** 2018-12-19

**Authors:** Sang-Tae Kim, Jae-Ryong Yoo, Jong Min Park

**Affiliations:** 1 Nuclear Emergency Division, Radiation Protection and Emergency Preparedness Bureau, Nuclear Safety and Security Commission, Seoul, South Korea; 2 Radiation Emergency Medicine Regulation Team, National Radiation Emergency Medical Center, Korea Institute of Radiological and Medical Sciences, Seoul, South Korea; 3 Department of Radiation Oncology, Seoul National University Hospital, Seoul, South Korea; 4 Institute of Radiation Medicine, Seoul National University Medical Research Center, Seoul, South Korea; 5 Biomedical Research Institute, Seoul National University Hospital, Seoul, South Korea; 6 Robotics Research Laboratory for Extreme Environments, Advanced Institutes of Convergence Technology, Suwon, South Korea; Northwestern University Feinberg School of Medicine, UNITED STATES

## Abstract

**Purpose:**

To investigate the internal exposure of nuclear medicine practitioners in South Korea.

**Methods:**

This study selected nuclear medicine practitioners among domestic hospitals and quantitatively measured their degree of internal exposure to radioisotopes, and conducted a dose assessment based on the results. For the dose assessment, 35 nuclear medicine practitioners at seven large hospitals were selected as the measurement subjects, and the measurements were obtained using the thyroid count, total body count, and a urine sample analysis. The internal exposure was measured once every two weeks, and measurements were obtained three to 15 times according to the practitioners.

**Results:**

As a result of measuring and analyzing the radionuclides with urine samples, one or more detections above the minimum detectable activity (MDA) was identified in 52 (15%) among all 340 cases for 14 of the practitioners (43%). The committed effective doses were evaluated as have a distribution of zero to 5.4 mSv, and were mostly 1 mSv or less. There were four practitioners exceeding 1 mSv based on the whole-body measurements, whose results from a urine sample analysis and thyroid monitoring all showed exposure of 1 mSv or less. All of the practitioners participated directly in the distribution and handling of radioactive sources, and none of the nurses exceeded 1 mSv. Furthermore, it was noteworthy that, among medical assistants who do not directly handle radioisotopes and are mainly involved in the transport of contaminated patients, there was one person who exceeded the whole-body measurement standard of 1 mSv.

**Conclusions:**

The committed effective dose of most nuclear medicine practitioners who participated in the survey was lower than 1 mSv. However, because the possibility of overexposure under special circumstances cannot be completely excluded, new strict radiation protection rules on the handling of open-source radioisotopes in hospitals are required for non-handling workers.

## Introduction

As the frequency and size of the diagnosis and treatment using radiation or radioisotopes have been increasing, the possibility of exposure of medical radiation workers has been increased. In particular, because nuclear medicine departments mainly use radioisotopes in the form of an open radioactive source, the possibilities of internal and external exposures to practitioners should be considered [1–3]. As of the end of November 2017, 109 nuclides have been licensed in 233 hospitals in the form of an open radioactive source, and 265 nuclides have been licensed from 329 organizations in the fields of industry, research, education, public affairs, and the military in South Korea. Furthermore, in South Korea, the number of radiation practitioners employed in hospitals has reached 5,100, and the total domestic use of I-131 (only for hospital use), which is subject to internal exposure control in hospitals, has reached 38,410 GBq in a total of 116 hospitals as of 2016. The total use in five out of the seven hospitals, subject to measurement in this study, were 1,303 to 5,005 GBq, which corresponds to the top-five domestic institutions in terms of I-131 use in South Korea [4] Moreover, liquid NaI-131 is mostly used alone and no sodium thiosulfate is added in South Korea, therefore, careful internal exposure control is required.

In South Korea, personnel who are concerned about internal exposure in accordance with the relevant public notifications are required to undergo personal monitoring of such exposure if the annual committed effective dose is expected to exceed 2 mSv [5]. Thus, nuclear power operators, nuclear fuel operators, and nuclear research institutions are routinely measuring and managing the internal exposures of workers who are concerned about internal exposure, whereas nuclear medicine practitioners in hospitals are not subject to the measurement or management of internal exposure. ICRP 130 [6], which provides stricter standards than domestic standards in South Korea, recommends that regular monitoring of internal exposure be performed if there is a risk of exceeding the committed effective dose of 1 mSv, and this standard has been under review for potential inclusion in South Korea.

For this study, seven nuclear medicine practitioners at large hospitals (approximately 500 to 3,000 beds) located in Seoul, the capital of South Korea, were selected, and the amount of internal radioisotope contamination in the body was measured to evaluate the dose based on the results. Because these hospitals are the top five domestic institutions in terms of I-131 use, they can represent the internal exposure dose of domestic hospitals from a stricter perspective.

This study can be used as basic data for the systematic management of internal exposure in hospitals by demonstrating and evaluating the internal exposure of nuclear medicine practitioners working at major hospitals in South Korea.

## Methods and materials

In this study, to evaluate the effects of radioisotopes present in the body of domestic hospital practitioners, 35 practitioners in the nuclear medicine department at seven large hospitals in Seoul (including the top-five domestic institutions in terms of I-131 use as of 2016) were measured during the period of October 30, 2015 to July 1, 2016 (an 8 month period). The practitioners selected are radiologists, nurses, medical assistants, and sanitation workers who are responsible for the distribution and handling of radioisotopes, intravenous injections, gamma camera scans, and others related operations. Although medical assistants and sanitation workers are not related to the treatment of radioisotopes, some of these workers were included as measurement subjects to check the presence or absence of radioactive contamination of radioisotope non-handling workers, who are in contact with radioisotope-administered patients or periodically access radioactively contaminated areas, and to infer the causes of such contamination.

Since the measurements were performed following the nuclear safety law by South Korea government (Nuclear Safety and Security Commission) for public health as well as all the data were anonymized, the institutional review board (IRB) gave exemption from reviews.

Although thyroid dosimetry is recommended for an internal dose assessment of I-131, if a thyroid measurement is infeasible, a bioassay method using urine samples is recommended in the IDEAS Guidelines ver. 2 [7]. Thus, both thyroid dosimetry and a urine sample analysis were conducted, including a whole-body measurement to identify the external contamination.

Because the results of a whole-body measurement cannot completely exclude the influence of an external contamination, the internal dose was evaluated by dividing the whole-body measurement results and the thyroid/urine sample measurement results. The method for collecting and measuring the urine samples is advantageous in that the method is relatively simple depending on the cooperation of the examinees, and because is possible to measure the alpha-, beta-, and low-energy gamma-emitting radionuclides without restraining the examinee during the measurements, which differs from a whole-body measurement. Urine samples were collected once ever 14 days, and 100 mL or more was collected three times to 15 times depending on the practitioner. After collecting the urine samples from the examinees, the urine samples were transferred to a gamma nuclide analysis laboratory for measurements.

Based on the measured results, Integrated Modules for Bioassay Analysis (IMBA) Professional Plus ver. 4.1.10 was used as a tool to evaluate the intake of radioisotopes as well as the committed effective dose. The IMBA computer program, which is commercial software that reflects the latest recommendations from the International Commission on Radiation Protection (ICRP), has been widely applied worldwide because it is easy to use, and makes it simple to evaluate the statistical errors and intakes of the measured samples [8].

### Whole-body measurements

Unlike I-131, which is the subject of this study, I-MIBG administered for the treatment of neuroblastoma in pediatric patients was evaluated as I_2_ because no residual excretory functions or internal dose coefficients were provided. The intake pattern was assumed as a single intake, and the absorption pattern was assessed as a vapor form (SR-1, ICRP 78) [7] at 1 mSv or more at the time of intake at the midpoint of the monitoring period (7 days). The time of intake was statistically re-evaluated using the content of the questionnaire as well as multiple measurements (assuming that no statistically significant difference was shown between the doses calculated with multiple measurements when the *p* value was 0.05 or higher), and the time of intake for the initial survey was determined based on the questionnaire. Measurements were conducted through a stand-type whole-body counter (Model RAD IQTM WBC, NuCare Inc., Incheon, South Korea) for 240 s per person, and immediately after the whole-body counting was completed, the thyroid measurement was obtained.

### Thyroid measurement and urine sample measurement

The thyroid measurement was conducted using a thyroid monitor which is a NaI detector (Model RAD IQTM Thyrowiz, NuCare Inc., Incheon, South Korea) for 240 s per person as done through whole-body counting. The instrument used for the urine sample measurement was a High Purity Germanium HP (Ge) detector (p-type HPGe detector model GC3018, Canberra, CT, USA), which has an excellent energy resolution and is accessible for analyzing nuclides; in addition, the measurement was performed for 1,800 s or more per sample. The time of intake was determined as the midpoint (day 7) of the monitoring period when detection occurred only in the thyroid measurement, as well as based on the questionnaire at the initial survey.

In the case of simultaneous detections from the thyroid and urine samples, the time of intake was estimated using the content of the questionnaire and statistical methods (assuming that there were no statistically significant differences between the doses calculated through multiple measurements when the *p* value was 0.05 or higher).

When a detection occurred only in the urine sample, the detection could be over-evaluated as 2,000 times the intake of the prior day if the intake was assumed as the midpoint (day 7) of the monitoring period, owing to the rapid decrease in urine excretion function after intake. The time of intake was determined by comparing the minimum detectable activity (MDA) values of the thyroid counter with the values of the urine samples.

Conditions other than the remaining intake point are the same as the evaluation method using the whole-body measurement results.

### Intake assessment

To evaluate the internal dose, it is essential to estimate the radionuclide intake from the measurement results obtained through direct or indirect measurement methods, as well as the residual and excretion functions. The *in vivo* residual function, which represents the residual amount in the body after a unit radioactivity intake, was used in the direct measurement method, and the *ex vivo* excretion function, which represents the *ex vivo* excretion rate, was used in the indirect measurement method, namely, Eq (1).

$$I=\frac{M}{m(t)}$$ (1)

where

*I*: radiation intake estimation (Bq);

*M*: measured value (Bq, Bq/d);

*m(t)*: *in vivo* residual function or *ex vivo* excretion function.

The thyroid remnant function and urine excretion function for I-131 are provided by ICRP 78 [1], which is appropriate for the chemical type of ingested nuclide such as the intake time, intake route, intake scenario, absorption pattern, and particle size.

### Internal exposure dose (committed effective dose) calculation

To manage the radiation exposure of workers and the general public for the purpose of radiological protection, the effective dose recommended by the ICRP was typically evaluated. Because the internal exposure is maintained over time after intake (50 years for adults, and 70 years for children), the internal exposure was evaluated using the committed effective dose. The committed effective dose is calculated as the product of the intake estimate and the internal dose conversion factor, namely, Eq (2).

$$E(50)=I∙e_{50}$$ (2)

where

*E(50)*: committed effective dose (Sv);

*e*_*50*_: internal dose conversion factor (Sv/Bq).

The currently used internal dose conversion factors for I-131 are provided in ICRP 78 [7] for radiation practitioners, and in ICRP 72 [9] for the public general; in addition, the values should be selected according to the intake route and chemistry type. In this study, we selected the Type F, Class SR-1, and dose conversion factor of 2×10^−8^ Sv/Bq.

## Results

In the present study, urine samples from the selected practitioners were collected and analyzed for radioactive nuclides. Tc-99m, I-123, I-131, Tl-201, and F-18 were detected in the urine samples. The committed effective dose, which was evaluated based on the measurement results of the urine samples, showed a distribution of zero to 5.4 mSv (I-131 only), but most were below the dose limit of 1 mSv. Because the concentration of Tl-201 nuclides in the body is very low, such contamination was excluded from the dose assessment. In the case of F-18, which is a short half-life nuclide emitting 0.511-MeV annihilation radiation, the committed effective dose of the nuclides could not be evaluated owing to the absence of the dose coefficient deposits. Thus, this study focused on the dose assessment results for I-131, which is the subject of internal exposure control in hospitals.

### Detection rate

The results were as follows: 1) the detection frequency above MDA in I-131 was 14 cases (43%) among 35 examinees in the seven institutions, and 52 cases (15%) among the 340 cases with a detection above the MDA, as shown in Table 1.

**Table 1:** Detection rate of I-131 relative to the examinees and examinations.

Hospital	Number of examinees			Number of examination		
	Total number of examinees	Examinee with one or more detections	Rate (%)	Total number of examinations a	Detection cases with above MDA	Rate (%)
A	2	2	100	34	16	47
B	4	3	75	56	12	21
C	6	3	50	57	6	11
D	14	3	21	125	12	9
E	4	1	25	46	1	2
F	3	1	33	14	2	14
G	2	1	50	8	3	38
Total	35	14	40	340	52	15

### Job analysis

Table 2 shows the detection details and work details of the thyroid and urine samples for each examinee.

**Table 2:** Detection and work details of thyroid and urine samples for each examinee.

Examinee code^a)^	Job type	Number of detection/examination	Chemical type	work details								
				Dispense	intravenous administration	Oral administration	Examination	Waste management	Safety management	patient Care	cleaning	Other RI handling
A-1	radiologic technologist	4/18	I_2_	√	√		√	√		√		
A-2	radiologic technologist	7/18	I_2_	√		√		√				
B-1	radiologic technologist	8/14	I_2_, I-MIBG	√	√	√		√				
B-2	radiologic technologist	2/7	I_2_			√		√				
B-3	radiologic technologist	0/18	I_2_	√	√	√	√	√	√	√		
B-4	radiologic technologist	0/18	non-handling^b)^									√
C-1	radiologic technologist	2/17	I_2_					√	√			
C-2	radiologic technologist	1/17	I_2_			√		√				
C-3	radiologic technologist	0/17	I_2_				√			√		
C-4	radiologic technologist	0/4	non-handling^b)^									√
C-5	nurse	0/4	non-handling^b)^									√
C-6	nurse	0/4	non-handling^b)^									√
D-1	sanitation worker	2/15	I_2_					√^c)^		√		
D-2	sanitation worker	4/15	I_2_								√	
D-3	nurse	1/15	I_2_		√			√		√		
D-4	radiologic technologist	1/15	I_2_	√		√						
D-5	sanitation worker	0/15	I_2_								√	
D-6	nurse	0/15	I_2_							√		
D-7	nurse	0/15	I_2_							√		
D-8	nurse	0/8	I_2_							√		
D-9	nurse	0/8	I_2_							√		
D-10	nurse	0/8	I_2_							√		
D-11	nurse	0/6	I_2_							√		
D-12	nurse	0/3	I_2_							√		
D-13	nurse	1/3	I_2_							√		
D-14	nurse	0/15	I_2_							√		
E-1	radiologic technologist	1/13	I_2_	√	√	√	√	√	√	√		
E-2	radiologic technologist	0/13	non-handling^b)^									√
E-3	radiologic technologist	0/13	non-handling^b)^									√
E-4	nurse	0/13	non-handling^b)^									√
F-1	radiologic technologist	2/5	I_2_			√	√			√		
F-2	radiologic technologist	0/5	I_2_	√	√	√	√			√		
F-3	radiologic technologist	0/5	non-handling^b)^									√
G-1	radiologic technologist	3/4	I_2_, I-MIBG	√	√	√		√		√		
G-2	nurse	0/4	I_2_							√		

a) The examinee code is represented as [institution code–examinee number].

b) Practitioners handling radioisotopes other than I-131 or I-MIBG.

c) Applicable to collection of clothes and bedding after patient use.

### Dose assessment using whole-body counter

Tables 3–8 shows the results of the dose assessment during the measurement period for each of the hospitals (A through G) as the measurement subject. Only the results regarding detections above MDA in the whole-body measurements based on the total examination order are shown in the table.

**Table 3:** Dose assessment results of whole-body measurement by institution A.

Practitioner	A-1					A-2				
Examination order	First	Second	Third	Tenth	Eleventh	First	Second	Tenth	Eleventh	Twelfth
Measurement results (Bq)	1111	5073	333	11264	2283	1107	423	6534	2721	919
Time of intake	2 days ago	0.2 days ago^a)^	No further intake	7 days ago	No further intake	2 days ago	No further intake	7 days ago	7 days ago	7 days ago
Intake (Bq)	4590	5127	-	33040	-	4574	-	44494	5816	4596
Committed effective dose(mSv)	0.091	0.101	-	0.654	-	0.091	-	0.881	0.115	0.091
Total dose (mSv)	0.846					1.178				

a) Using multiple measurement results from 2nd and 3rd order (*p* = 0.173)

**Table 4:** Dose assessment results of whole-body measurement by institution B.

Practitioner	B-1							B-2		B-3
Examination order	Third	Fourth	Seventh	Eleventh	Twelfth	Thirteenth	Fourteenth	First	Second	First
Measurement results (Bq)	1150	217	910	87405	6433	2877	24509	179	1132	2130
Time of intake	7 days ago	no further intake	7 days ago	0.1 days ago^a)^	no further intake	7 days ago	7 days ago	1 day ago	7 days ago	1 day ago
Intake (Bq)	7831	-	6197	87640	-	6449	164800	525	7354	6100
Committed effective dose(mSv)	0.155	-	0.123	1.735	-	0.128	3.263^b)^	0.010	0.146	0.121
Total dose (mSv)	5.404							0.156		0.121

a) Using multiple measurement results of 11th and 12th orders (*p* = 0.619).

b) Because the work ceased after the 14th examination, no further examinations were conducted. In addition, because the daily work was repeated (questionnaire), 3.19 mSv was assumed as chronic intake.

**Table 5:** Dose assessment results of whole-body measurement by institution C.

Practitioner	C-1	C-2		C-3
Examination order	Fourth	Third	Fourth	Third
Measurement results (Bq)	292	2907	426	136
Time of intake	7 days ago	1 day ago^a)^	no further intake	7 days ago
Intake (Bq)	1988	7099	-	926
Committed effective dose(mSv)	0.039	0.140	-	0.018
Total dose (mSv)	0.039	0.140		0.018

a) Using multiple measurement results of third and fourth orders (*p* = 0.156)

**Table 6:** Dose assessment results of whole-body measurement by institution D.

Practitioner	D-1				D-2
Examination order	11-1^st^	11-2^nd^	Twelfth	Thirteenth	Second
Measurement results (Bq)	7735	5606	1595	375.05	193
Time of intake	4 days ago	no further intake	7 days ago	no further intake	7 days ago
Intake (Bq)	44090	-	10860	-	1314
Committed effective dose(mSv)	0.873	-	0.215	-	0.026
Total dose (mSv)	1.088				0.026

**Table 7:** Dose assessment results of whole-body measurement by institution E.

Practitioner	E-1
Examination order	Tenth
Measurement results (Bq)	19697
Time of intake	1 day ago^a)^
Intake (Bq)	57770
Committed effective dose(mSv)	1.144
Total dose (mSv)	1.144

a) Based on the contents of the questionnaire, the examination was completed after the tenth order.

**Table 8:** Dose assessment results of whole-body measurement by institution G.

Practitioner	G-1		
Examination order	First	Third	Fourth
Measurement results (Bq)	34507	11973	1225
Time of intake	2 days ago	0.2 days ago^a)^	no further intake
Intake (Bq)	142600	15120	-
Committed effective dose(mSv)	2.823	0.299	-
Total dose (mSv)	3.123		

a) Using multiple measurement results of the third and fourth orders (*p* = 0.726)

For hospital F, no detections were above MDA in the whole-body measurements. The IDEAS level evaluation of whole-body measurement results is shown in Table 9.

**Table 9:** IDEAS level evaluation of whole-body measurement results.

Level	Dose	Number of measurements
Level 0	D < 0.1 mSv/y	307
Level 1	0.1 mSv/y < D < 1 mSv	29
Level 2	1 mSv < D < 6 mSv	4
Level 3	6 mSv < D	0
Total		340

The annual dose was calculated by dividing the total dose during the examination period (mSv) by the examination period (8 months) and multiplying it by 12 (number of months in a year). The results are shown in Table 10.

**Table 10:** Annual dose assessment for whole-body measurement results.

Institution	practitioner	total dose during the examination period (mSv)	Period (month)	Annual dose (mSv/y)
A	A-1	0.846	8	1.269
	A-2	1.178	8	1.767
B	B-1	5.404	6	10.808
	B-2	0.156	3	0.624
	B-3	0.121	8	0.182
C	C-1	0.039	8	0.059
	C-2	0.140	8	0.210
	C-2	0.018	8	0.027
D	D-1	1.088	8	1.632
	D-2	0.026	8	0.039
E	E-1	1.144	6	2.288
G	G-1	3.123	2	18.738
Average		1.107	6.8	3.137

### Dose assessment using measurement results from thyroid and urine samples

As in the whole-body measurement results, only the results above MDA for the thyroid and urine sample measurements based on the total examination order are shown in Tables 11–17. The assessment of IDEAS level evaluation for thyroid and urine measurement results is shown in Table 18. The annual dose assessment for thyroid and urine measurement results is shown in Table 19.

**Table 11:** Dose assessment results from thyroid and urine sample measurements by institution A.

Practitioner		A-1				A-2						
Examination order		Sixth	Tenth	Eleventh	Eighteenth	Fourth	Seventh	Tenth	Eleventh	Twelfth	Fourteenth	Seventeenth
Measurement results	thyroid (Bq)	<MDA	<MDA	<MDA	<MDA	<MDA	<MDA	<MDA	<MDA	<MDA	<MDA	<MDA
	urine (Bq/d)	9	129	3	11	25	13	234	30	12	20	60
Time of intake		3 days ago	2 days ago	3 days ago	3 days ago	2 days ago	2 days ago	1.5 days ago	2.5 days ago	3 days ago	2.5 days ago	2 days ago
Intake (Bq)		2937	2515	53	3573	488	261	451	2368	3656	1638	1176
Committed effective dose(mSv)		0.058	0.050	0.001	0.071	0.010	0.005	0.009	0.047	0.072	0.032	0.023
Total dose (mSv)		0.180				0.199						

**Table 12:** Dose assessment results from thyroid and urine sample measurements by institution B.

Practitioner		B-1								B-2	
Examination order		Third	Fourth	Seventh	Ninth	Tenth	Eleventh	Twelfth	Fourteenth	First	Second
Measurement results	thyroid (Bq)	<MDA	740	<MDA	<MDA	<MDA	485	<MDA	<MDA	<MDA	<MDA
	urine (Bq/d)	24	45	19	10	574	88	14	17	21	62
Time of intake		2.5 days ago	2.5 days ago^a)^	2.5 days ago	3 days ago	2.5 days ago	2 days ago^b)^	3 days ago	2.5 days ago	2.5 days ago	2 days ago
Intake (Bq)		1935	3483	1549	3297	1107	2107	4504	1365	1709	1207
Committed effective dose(mSv)		0.038	0.071	0.031	0.065	0.022	0.042	0.089	0.027	0.034	0.024
Total dose (mSv)		0.385								0.058	

a) Using multiple measurements of thyroid/urine samples (p = 0.940)

b) Using multiple measurement results of thyroid/urine samples (p = 0.633)

**Table 13:** Dose assessment results from thyroid and urine sample measurements by institution C.

Practitioner		C-1		C-2
Examination order		First	Sixth	Third
Measurement results	thyroid (Bq)	<MDA	<MDA	<MDA
	urine (Bq/d)	9	4	14
Time of intake		3 days ago	3 days ago	3 days ago
Intake (Bq)		2876	1404	4667
Committed effective dose(mSv)		0.057	0.028	0.092
Total dose (mSv)		0.085		0.092

**Table 14:** Dose assessment results from thyroid and urine sample measurements by institution D.

Practitioner		D-1			D-2				D-3	D-13
Examination order		Sixth	11-1th	11-2th	Sixth	Seventh	Thirteenth	Fourteenth	Sixth	Third
Measurement results	thyroid (Bq)	<MDA	1274	1108	<MDA	<MDA	<MDA	<MDA	<MDA	<MDA
	urine (Bq/d)	41	109	14	128	12	53	14	25	5
Time of intake		2.5 days ago	2 days ago^a)^	No further intake	2 days ago	3 days ago	2.5 days ago	3 days ago	2.5 days ago	3 days ago
Intake (Bq)		3291	5404	-	2500	3917	4255	4635	2007	380
Committed effective dose(mSv)		0.0651	0.149	-	0.050	0.078	0.084	0.092	0.040	0.008
Total dose (mSv)		0.149			0.303				0.040	0.008

a) Using multiple measurement results of 11-1th and 11-2th thyroid/urine samples (*p* = 0.647)

**Table 15:** Dose assessment results from thyroid and urine sample measurements by institution E.

Practitioner		E-1
Examination order		Tenth
Measurement results	thyroid (Bq)	918
	urine (Bq/d)	3074
Time of intake		1 days ago^a)^
Intake (Bq)		4256
Committed effective dose(mSv)		0.084
Total dose (mSv)		0.084

**Table 16:** Dose assessment results from thyroid and urine sample measurements by institution F.

Practitioner		F-1	
Examination order		First	Third
Measurement results	thyroid (Bq)	<MDA	<MDA
	urine (Bq/d)	29	17
Time of intake		2.5 days ago	2.5 days ago
Intake (Bq)		2344	1397
Committed effective dose(mSv)		0.046	0.028
Total dose (mSv)		0.074	

**Table 17:** Dose assessment results from thyroid and urine sample measurements by institution G.

Practitioner		G-1		
Examination order		First	Third	Fourth
Measurement results	thyroid (Bq)	566	287	<MDA
	urine (Bq/d)	<MDA	140	36
Time of intake		2 days ago	2 days ago^a)^	2.5 days ago
Intake (Bq)		2538	1420	2818
Committed effective dose(mSv)		0.050	0.028	0.056
Total dose (mSv)		0.134		

a) Using multiple measurements of thyroid/urine samples (*p* = 0.135)

**Table 18:** Assessing IDEAS level evaluation for thyroid and urine measurement results.

Level	Dose	Number of measurements
Level 0	D < 0.1 mSv/y	301
Level 1	0.1 mSv/y < D < 1 mSv	39
Level 2	1 mSv < D < 6 mSv	0
Level 3	6 mSv < D	0
Total		340

**Table 19:** Annual dose assessment for thyroid and urine measurement results.

Institution	practitioner	total dose during the examination period (mSv)	Period (month)	Annual dose (mSv/y)
A	A-1	0.180	8	0.270
	A-2	0.199	8	0.299
B	B-1	0.385	6	0.770
	B-2	0.058	3	0.232
C	C-1	0.085	8	0.128
	C-2	0.092	8	0.138
D	D-1	0.149	8	0.224
	D-2	0.303	8	0.455
	D-3	0.040	8	0.060
	D-13	0.008	1	0.096
E	E-1	0.084	6	0.168
F	F-1	0.074	2	0.444
G	G-1	0.134	2	0.804
Average		0.138	5.8	0.314

## Discussions

In the classification of job types for practitioners at hospitals, no nurses showed a dose exceeding 1 mSv, whereas some of the radiologic technologists who participate directly in the distribution of radioisotopes showed a dose exceeding 1 mSv. It is particularly noteworthy that, among medical assistants D, who do not deal directly with radioisotopes but are mainly in physical contact with contaminated sources such as in the transport of radioisotope-administrated patients, as well as the collection of contaminated patient clothing and bedding, there was one person who exceeded a dose of 1 mSv based on the whole-body measurement standard. However, although I-131 was detected in the whole-body measurement result of this particular medical assistant, I-131 was not detected in the thyroid or urine measurements. This result suggests that, when transferring radioisotope-administrated patients, contact with their contaminated clothing, saliva, or nasal discharge, or a combination thereof, could have affected the medical assistant. Thus, the results indicate that, despite the absence of direct internal exposure, indirect internal exposure can occur.

In the case of D-2, who are responsible for sanitation, radioactivity above MDA was not detected in the whole-body measurements, whereas radioactivity above MDA was detected in the urine measurements at 1 mSv or less. In this regard, sanitation workers who periodically access and collect garbage from contaminated laboratories can be classified as potentially the most vulnerable in terms of protection from internal exposure, as compared to medical assistants.

Although internal exposure would be less likely to occur through the patients than in directly dealing with I-131, the staff are responsible for the transport of, and are in frequent contact with, radioisotope-administered patients; in addition, sanitation workers periodically access contaminated areas and are continuously exposed to contamination. Thus, it is essential to establish pollution prevention procedures and educate the staff and sanitation workers. Previous radiation exposure management has focused on radiation workers who are directly handling radiation generating devices or radioisotopes. However, the results suggest that radiation protection measures should be separately established for non-radiation workers such as medical assistants or sanitation workers who periodically access a radiation management area, and are directly exposed to contaminant sources (patients and/or waste).

Because a whole-body measurement result cannot be completely excluded from the effects of external contamination, an internal dose assessment was conducted by dividing the whole-body measurement results and the thyroid/urine sample measurement results. As a result, the job analysis results for the examinees with a high detection frequency showed a high possibility of internal contamination while dispensing work handling solution (liquid) forms. In particular, very frequent internal contamination occurred in workers handling I-MIBG, which is used for neuroblastoma screening in children (B-1 and G-1 in Table 2). This observation can be explained based on the characteristics of children who become more agitated during a diagnosis than adults, owing to contamination during the I-MIBG injection process. In addition, although dispensing of I-MIBG is relatively complicated, B-1 and G-1 workers were not skillful owing to their little experience, which resulted in a long handling time. Therefore, an appropriate skills training for workers is of necessity for contamination prevention. In other work details, there are no clear correlations between the work details and detection details, and it is thought that the degree of internal exposure varies depending on the environment of the work facility, skill levels of the individual workers, and the protection efforts of the individual workers. However, B-1 and G-1 workers, who were evaluated as being exposed to an annual dose of 10 mSv, were evaluated by regarding their chemical type as I_2_ in spite of handling I-MIBG. Thus, for a more accurate dose assessment, a dose re-evaluation is required using the information on the physiological behavior of I-MIBG in the future.

Although I-131 is usually in the form of an open radioactive source in hospitals, it is mainly obtained and used in capsule form, with some are supplied in solution form; thus, there would be no workers whose radiation doses are evaluated as abnormally high because many workers alternately perform open radioactive source distributions in most hospitals.

Although urine samples were collected in the form of spot samples in this study, the collection of samples over a 24-h period is required to evaluate the dose more precisely. After establishing the relationship between the spot samples and 24-h samples, and deriving the errors, it will be further necessary to consider how appropriate the amount of the representative 24-h sample is, as estimated from the spot samples. Moreover, it is necessary to derive the correlation between airborne concentration data and dose assessment by comparing the airborne concentration data with the dose assessment results at the corresponding hospital. Furthermore, a more accurate dose assessment can be available if urine samples are collected and analyzed for more than 1 year to estimate the intake according to the seasonal factors when considering the degree of indoor ventilation.

## Conclusions

The committed effective dose of most nuclear medicine practitioners who participated in the survey was lower than 1 mSv, as provided in ICRP 130 [4]. These results indicate that most of the nuclear medicine practitioners do not need regular monitoring of their internal exposure (body contamination) to radionuclides, and the health effects by such exposure are at an insignificant level. However, because the possibility of overexposure under special circumstances cannot be completely excluded, new strict radiation protection rules on the handling of open-source radioisotopes in hospitals are required for non-handling workers such as medical assistants and sanitation workers, as well as for radiation practitioners. It is of particular necessity to ensure that the distribution work not be placed into the hands of a single person, to improve the ventilation of the facilities, and to protect those practitioners who do not need to handle the corresponding radioactive sources from exposure by storing the various types of open radioactive sources in a single location.

Furthermore, because workers handling I-MIBG, which is used for neuroblastoma screening in children, showed the highest level of internal exposure, I-MIBG handling (particularly injections) requires special procedures and skills training for contamination prevention. It will be further necessary to periodically measure the working environment or frequently monitor the concentration of radionuclides in the air to prevent or reduce the possibility of internal exposure.
